# Advances in Primary Progressive Aphasia

**DOI:** 10.3390/brainsci12050636

**Published:** 2022-05-12

**Authors:** Jordi A. Matias-Guiu, Robert Laforce, Monica Lavoie, Rene L. Utianski

**Affiliations:** 1 Department of Neurology, Hospital Clínico San Carlos, Health Research Institute “San Carlos” (IdISCC), Universidad Complutense de Madrid, 28040 Madrid, Spain; 2 Clinique Interdisciplinaire de Mémoire, Département des Sciences Neurologiques du CHU de Québec, Faculté de Médecine, Université Laval, Quebec City, QC G1V 0A6, Canada; robert.laforce@fmed.ulaval.ca (R.L.J.); monica.lavoie.1@ulaval.ca (M.L.); 3 Department of Neurology, Mayo Clinic, Rochester, MN 55902, USA; utianski.rene@mayo.edu

Primary progressive aphasia (PPA) is a neurodegenerative syndrome characterized by progressive and predominant language impairment. Our knowledge of this disorder has evolved significantly in recent years. Notably, correlations between clinical findings and pathology have improved, and main clinical, neuroimaging, and genetic features have been described. However, as in other neurodegenerative syndromes, diagnosis is often challenging, a better understanding of natural history is needed, and successful therapies are lacking.

In this context, this Special Issue of *Brain Sciences* is focused on “Advances in Primary Progressive Aphasia (PPA)” and includes articles addressing advances in the diagnosis, expected progression, and treatment of PPA, each of which is elucidated further in what follows.

## 1. Diagnosis

In “Contribution of the Cognitive Approach to Language Assessment to the Differential Diagnosis of Primary Progressive Aphasia” [1], the authors reviewed the contribution of the assessment of specific language abilities in the differential diagnosis of PPA, the main cognitive processes involved in each task, and the findings supportive of each variant. 

In “Breakdowns in Informativeness of Naturalistic Speech Production in Primary Progressive Aphasia” [2], the authors examined 101 participants, including 70 patients with a diagnosis of PPA (19 svPPA patients, 26 lvPPA patients, and 25 nfvPPA) and 31 age-matched controls, using the “Picnic Scene” from the Western Aphasia Battery-Revised. The informativeness of speech was quantified. Relative informativeness, or efficiency, of speech was preserved in non-fluent variant PPA patients and impaired in logopenic and semantic variants. These findings support the value of assessing and quantifying functional communication in PPA, which could be useful in the diagnosis and complement other parameters of spontaneous speech analysis. 

In “Verbal Short-Term Memory Disturbance in the Primary Progressive Aphasias: Challenges and Distinctions in a Clinical Setting” [3], the authors examined short-term memory profiles in four well-characterized patients with lvPPA, nfvPPA, svPPA, and Alzheimer’s disease. The authors also discussed the adequate tasks to evaluate short-term memory, the influence of other cognitive functions, and the relevance of examining visuospatial short-term and working memory. 

In “Primary Progressive Aphasia: Use of Graphical Markers for an Early and Differential Diagnosis” [4], the authors analyzed writing pressures during linguistic and non-linguistic tasks in patients with PPA, Alzheimer’s disease, and healthy controls. Several differences were found between groups depending on the type of task. 

Two studies examined the application of electroencephalography (EEG) in the diagnosis of PPA and its variants. In “Application of Machine Learning to Electroencephalography for the Diagnosis of Primary Progressive Aphasia: A Pilot Study” [5], a cross-sectional study with 40 PPA patients and 20 controls was performed. Patients underwent resting-state EEG. Several data extraction procedures were performed, and several machine learning algorithms were evaluated. Diagnostic capacity was relatively high for the discrimination between PPA and controls, while the classification between variants was lower. The most important features in the classification were those derived from network analysis based on graph theory. In this regard, in the study “A Preliminary Report of Network Electroencephalographic Measures in Primary Progressive Apraxia of Speech and Aphasia” [6], the authors evaluated EEG changes in the agrammatic PPA and primary progressive apraxia of speech variants using graph theory analysis. Several network alterations were found, and interestingly, there were correlations between EEG graph theory measures and certain clinical measures. Overall, both studies suggest the potential for further application of EEG in PPA. 

## 2. Longitudinal Change

In “Longitudinal Changes in Cognition, Behaviours, and Functional Abilities in the Three Main Variants of Primary Progressive Aphasia: A Literature Review” [7], the authors provided a literature review on the natural history of the three main variants of PPA. The review focused on cognitive, behavioral, and functional changes associated with the syndromes. Findings from 15 studies included in the review showed that the svPPA was associated with more behavioral disturbances both at baseline and over the course of the disease, whereas lvPPA experienced worse cognitive decline and faster progression to dementia. The most significant decline in language production and functional abilities was found in individuals with nfvPPA. This review highlighted the need for more data on lvPPA, surprisingly, given it is the most frequent subtype of PPA. The authors also reported a lack of data regarding the prodromal and last stages of PPA, which could be very helpful for patients and families.

In “Survival in the Three Common Variants of Primary Progressive Aphasia: A Retrospective Study in a Tertiary Memory Clinic” [8], the authors analyzed survival data in a cohort of 83 deceased patients with a diagnosis of PPA. They reported a significantly longer survival from symptom onset and diagnosis in svPPA than in the two other variants. Indeed, the mean survival from symptom onset was 7.6 years for lvPPA, 7.1 years for nfvPPA, and 12 years for svPPA. The most common causes of death were natural cardio-pulmonary arrest and pneumonia. These findings provide invaluable data to healthcare professionals, as well as patients and families, about the progression of the disease and the end stages of life.

## 3. Treatment

In “Treatment for Anomia in Bilingual Speakers with Progressive Aphasia” [9], the authors explored the impact of the lexical retrieval cascade treatment approach on a group of bilingual patients with heterogeneous clinical presentations, including right-sided temporal frontotemporal dementia and semantic and logopenic PPA. Overall, participants demonstrated a significant treatment effect in each of the targeted languages and showed a cross-linguistic transfer for trained cognates in both languages that were maintained up to one-year post-treatment. While there was a decline in clinical measures of language and cognition, patient and care partner reported outcomes indicated communication was “somewhat better.” The findings of the study support the important conclusions that (1) monolingual clinicians may be able to select cross-linguistic cognates as a means to support gains across languages, even for words trained in a single language, and (2) the importance of including patient-reported outcome measures in intervention studies.

In “Cognitive Intervention Strategies Directed to Speech and Language Deficits in Primary Progressive Aphasia: Practice-Based Evidence from 18 Cases” [10], the authors demonstrated the importance of symptom-targeted intervention in a group of patients with PPA, again with heterogeneous clinical presentations. While there was no control cohort or within-group comparison to determine the impact of alternative treatment approaches, the patients showed improved performance in trained items at post-test, with an individualized focus on either naming, sentence production, motor speech functioning, or phonological functioning. While 18 patients completed their personalized treatment, this was 56% of those recruited; it is also important to note that 25% of patients recruited did not complete the intervention because of frustration, anxiety, motivation, or other practical–logistical barriers. Many of the participants who completed the program had an objective decline in function at follow-up but reported subjective improvement that was not otherwise quantified, again highlighting the importance of patient-reported outcomes. Overall, the study supports the potential for symptom-targeted intervention and suggests that completing this in the early stages of the disease may improve adherence and the subsequent possibility of positive treatment outcomes. 

In “Semantic Variant Primary Progressive Aphasia: Practical Recommendations for Treatment from 20 Years of Behavioural Research” [11], the authors highlighted the different sources of word-finding difficulties in PPA, including impairment in semantic knowledge in semantic, lexical access, and phonological impairment in logopenic, and post-lexical execution in non-fluent/agrammatic. With the focus on semantic PPA, they discussed the important implications of left and right atrophy, where patients with right-sided temporal atrophy may have greater behavioral changes, loss of insight, and altered pragmatics. It is crucial to recognize that these non-aphasic cognitive-communication challenges are well within the scope of speech pathology to address in intervention. In this review, the authors discussed the outcomes of different naming treatments and the benefit of capitalizing upon preserved long-term memory systems. They noted that in maintenance or compensatory approaches, the severity of impairment should inform the nature of the intervention. For instance, patients may not be able to learn how to augmentative or alternative communication devices later in the disease, so it is important to incorporate this before the skills to acquire its use are lost. They also mentioned the benefit of interdisciplinary treatment, with collaboration between speech and occupation therapy, and focusing on activities of daily living. Finally, they discussed the benefit of education and support group programs as a safe forum for discussing experiences and sharing resources and strategies, further highlighting the needs of both patient and care partners should both be addressed, simultaneously.

## 4. Conclusions

Taken together, the papers in this Special Issue, addressing the diagnosis, treatment, and expected progression of PPA, contribute to the literature and our understanding of this heterogeneous patient population. We strongly believe that speech–language pathologists, neurologists, and neuroscientists alike will benefit from the original research studies and reviews amassed in this collection, and as the Guest Editors of this Special Issue, we thank the authors for their contributions.

## Data Availability

Not applicable.
